# Rapid construction of a whole-genome mutant library by combining haploid stem cells and inducible self-inactivating PiggyBac transposon

**DOI:** 10.1007/s13238-020-00702-0

**Published:** 2020-03-19

**Authors:** Junjie Mao, Kai Xu, Jiabao Han, Guihai Feng, Ying Zhang, Wei Li

**Affiliations:** 1 State Key Laboratory of Stem Cell and Reproductive Biology, Institute of Zoology, Chinese Academy of Sciences, Beijing, 100101 China; 2 Institute for Stem Cell and Regeneration, Chinese Academy of Sciences, Beijing, 100101 China; 3 University of Chinese Academy of Sciences, Beijing, 100049 China

**Dear Editor,**

Genome-scale screening is a powerful method used to explore phenotypes that are of interest, and numerous screening systems have been built for functional sites identification. Classical genetic screening in eukaryotes has been performed using chemical mutagens (Chen et al., 2000), transposon mediated gene trapping (Dupuy et al., 2005), and CRISPR-Cas9 system (Wang et al., 2014). These have helped us to decipher many biological processes. Although numerous screening methods have been developed presently (Schneeberger, 2014), most of them are time consuming and expensive for library designing, preparing, and screening. In addition, classical screening systems reveal target genes with high false positive rates because it is very difficult to ensure that only one target is disrupted in a single cell. These disadvantages, which commonly exist in typical genome-wide screening, hinder the widespread application of forward genetic screening.

In previous reports, PiggyBac (PB) transposon was performed for insertional mutagenesis in mammals (Wang et al., 2009). Recently, a report has been developed about “Slingshot” screening method, a self-inactivating PB transposon system for tamoxifen inducible insertional mutagenesis (Kong et al., 2010). While Slingshot screening system has been used for gain-of-function, loss-of-function, and genome-wide screens, it is difficult to ensure only one insertional mutagenesis in one cell because of the multi-copy Slingshot vector entry.

Here, we have developed a new screening system named “One-Shot” which is based on Slingshot method and haploid embryonic stem cells (haESCs) (Li et al., 2012). In “One-Shot” screening system, we integrated PiggyBac based gene trapping cassette into *mRosa26* locus via CRISPR-Cas9 assisted homologous recombination in mouse haESCs, and we ensured no random insertion with thymidine kinase (TK) negative selection (Fig. 1A). By design, correctly integrated clones have only one copy of trapping cassette and characteristic of drugs resistance (Fig. S1A and S1B). Furthermore, without the induction of 4-hydroxytamoxifen (4-OHT), “One-Shot” haESCs can be cryopreserved and expanded homogeneously. Since “One-Shot” system was generated, screening could be initiated by supplementing 4-OHT into the medium. This enabled PB transposase-oestrogen receptor ligand-binding domain complex (PBase-ERT2) transport into the nucleus and induced transposition (Fig. 1B). In addition, PB transposase (PBase) in “One-Shot” was self-inactivated after 4-OHT treatment to prevent re-transposition of trapping element (Fig. 1C). From the data, “One-Shot” in haESCs was integrated in the proper position and expressed spatiotemporally.

**Figure 1:**
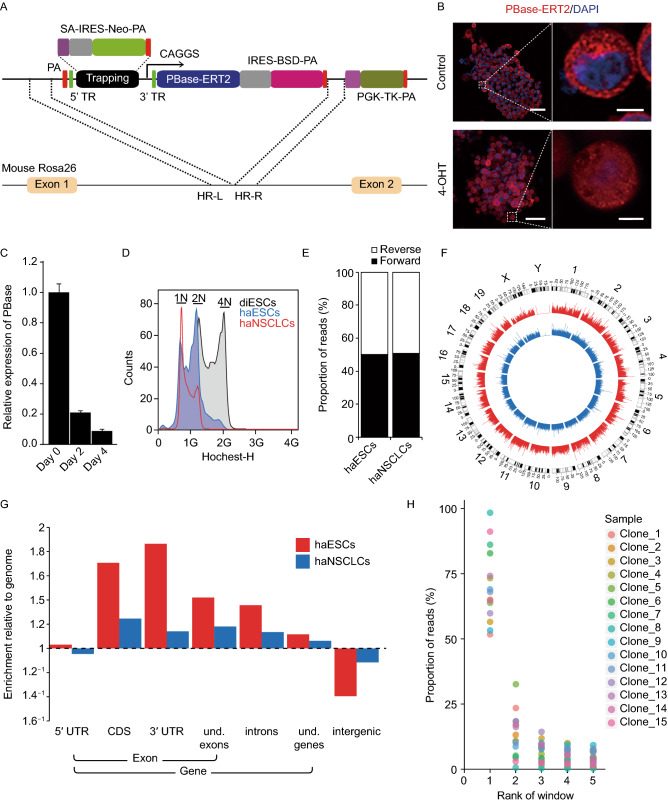
**Generation of “One-Shot” system in haploid cells**. (A) Schematic diagram of genetic screening system for mutation of one site per cell. The system contains trapping cassette, self-inactivating PBase, TK negative selection element, and homologous recombination arms. With the help of CRISPR-Cas9, one copy of the screening cassette can be integrated into *mRosa26* locus of haploid embryonic stem cells, leading to one trapping cassette for each cell, which we identified as “One-Shot”. PA, poly A signal; SA, splice accepter; IRES, internal ribosome entry site; Neo, neomycin resistant gene; TR, terminal repeat; ERT2, oestrogen receptor ligand-binding domain; PBase, PiggyBac transposase; BSD, blasticidin; TK, thymidine kinase; HR-L, homologous recombination left arm; HR-R, homologous recombination right arm. (B) Spatial-specific expression of PBase with (bottom) or without (top) 4-hydroxytamoxifen (4-OHT) treatment. 4-OHT bound to the PBase-ERT2 transporting it into the nucleus. PBase-ERT2 was marked red, nuclei were stained with DAPI. Scale bars are 50 μm (left-hand panels) and 5 μm (right-hand panels). (C) Quantitative real-time polymerase chain reaction (qRT-PCR) analysis of temporal expression levels of PBase, “One-Shot” haploid embryonic stem cells (haESCs) were treated with 4-OHT for 2 days and 4 days. This inactivated PBase by excising promoter “CAGGS” from it after translocation of trapping cassette. (D) Flow cytometry analysis of DNA content of “One-Shot” haploid neural stem cell-like cells (haNSCLCs). “One-Shot” haNSCLCs were differentiated from “One-Shot” haESCs with the protocol we described previously (He et al., 2017). (E) Proportion of forward and reverse strands covered by reads; reads that came from “One-Shot” haESCs and “One-Shot” haNSCLCs were aligned to reference genome with mapping quality higher than 30. For haESCs, proportion of reads located in forward strand was 50.0272%; and for haNSCLCs, the proportion was 50.6362%. (F) Multiplex circular graph showing insertion sites of “One-Shot” haESCs (red) and “One-Shot” haNSCLCs (blue). Inserted sites were isolated from coverage assay, which treated 4 × 10^7^ “One-Shot” haESCs and “One-Shot” haNSCLCs with 4-OHT for 3 days respectively. (G) Reads from “One-Shot” haESCs and “One-Shot” haNSCLCs were counted, and the enrichment of reads relative to genome from each feature was showed via histogram. Feature enrichment score = (reads in a feature/total reads)/(length of feature/genome size). (H) 15 clones were derived from transposed “One-Shot” haESCs using flow cytometry. For each clone, reads from inserted sites were counted by the window of 100,000 bp. After sorting by read counts for each window, top 5 windows (x-axis) and the corresponding proportion of reads (y-axis) were shown using scatter plots

Because haESCs can be differentiated with haplotype (He et al., 2017), we differentiated “One-Shot” haESCs into haploid neural stem cell-like cells (haNSCLCs) to obtain “One-Shot” haNSCLCs (Figs. 1D and S1C), which broaden the scope of application of “One-Shot” system.

To evaluate the footprints of “One-Shot” in genome, we expanded “One-Shot” haESCs and “One-Shot” haNSCLCs in a population of 4 × 10^7^ respectively, and then added 4-OHT to trigger the transposition events. After 3 days of 4-OHT induction, cells were harvested to amplify the inserted sites using splinkerette PCR (Horn et al., 2007). Next, PCR products from “One-Shot” haESCs and “One-Shot” haNSCLCs were sequenced separately by next generation sequencing. As expected, PiggyBac mediated transpositions were distributed both in forward and reverse strands of genome without phenotype screening (Fig. 1E) and footprints of trapping element in “One-Shot” system were distributed in the whole genome (Fig. 1F). From global overview of reads distribution, we found that PB transposon could insert in many genome features. Consistent with previous report, we observed the preference of integration of this system within transcription units (Fig. 1G) (Ding et al., 2005). Furthermore, we picked 15 clones from the transposed “One-Shot” haESCs library using flow cytometry, and inserted sites for each clone were isolated via barcodes encoded splinkerette PCR and high throughput sequencing. With our expectation, only one inserted site was identified from most of the clones (Fig. 1H). Although it was very difficult to completely self-inactivate PBase in “One-Shot” haESCs (Fig. 1C), most of reads were located in first inserted site in these clones (Fig. 1H). For this part, we evaluated the coverage of “One-Shot” system and found that it could be inserted in whole genome.

To evaluate the feasibility of “One-Shot” screening system, we explored the genes that delay exit from naïve state using “One-Shot” haESCs withdrawal of 2i and LIF. To monitor the process of differentiation, destabilized GFP expressed from endogenous *Rex1* (*Zfp42*) locus was generated in “One-Shot” haESCs (“One-Shot” *Rex1-*GFP haESCs, OsRG-haESCs) (Wray et al., 2011). To validate the sensitivity, we first differentiated OsRG-haESCs in N2B27 medium without 2i and LIF, and observed few OsRG-haESCs retained GFP positive within 7 days (Fig. S2A). We next prepared OsRG-haESCs for naïve pluripotency exit screening. At the beginning, OsRG-haESCs were purified by flow cytometry to confirm that more than 90% cells were haploid. Next, these cells were divided into 3 libraries, and each library contained 1.5 × 10^7^ cells, which were cultured in N2B27 medium without 2i and LIF for random differentiation. After 32 days, GFP positive cells from 3 libraries were collected respectively (Fig. 2A). GFP-positive cells from two libraries were sequenced using high throughput sequencing to isolate the trapped genes directly (library OSD_1 and library OSD_2), and GFP positive cells from the rest of library (library OSD_B) were plated onto dish with feeders and cultured in N2B27 medium with 2i and LIF for 7 days before isolating inserted sites.

**Figure 2:**
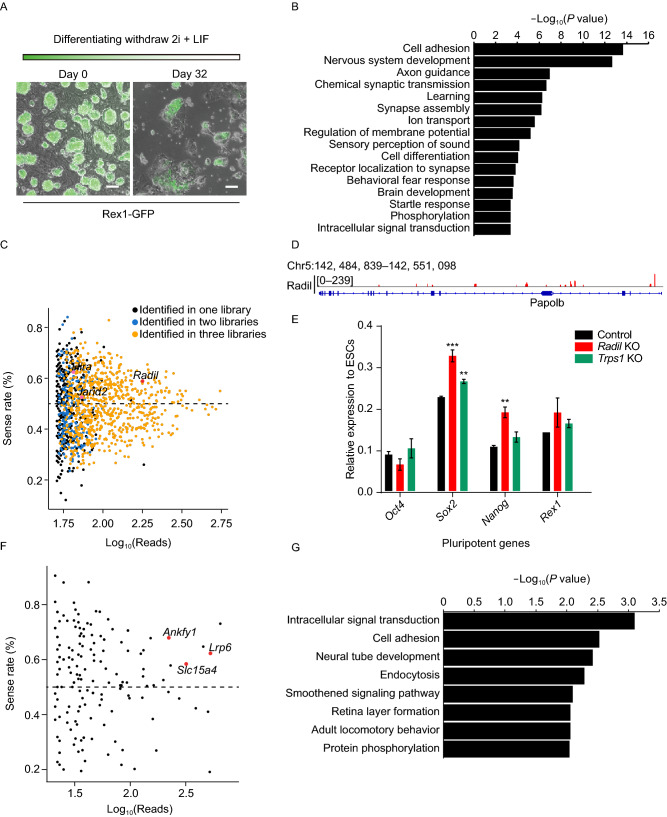
**Application of “One-Shot” system in ESCs self-renewal and puromycin screening**. (A) Rex1-GFP expression in “One-Shot” haESCs in 2i/LIF (left) and after differentiation for 32 days in N2B27 (right) was detected by fluorescence microscope, scale bar, 100 μm. (B) Gene ontology (GO) analysis of trapped genes from self-renewal screening. (C) Insertion orientation relative to affected genes was distinguished and counted, and inserted sites with the same orientation of genes were effective for trapping. For each gene, the sense rates of insertions (y-axis) and the total number of reads for each insertions (x-axis) are shown in scatter plots. Recovered frequency of genes which were identified in three independent libraries was distinguished with colors. Genes previously implicated in maintenance of pluripotency of ESCs and appeared in three libraries were marked with pink plots, and *Radil* was highlighted with a red plot. (D) Read enrichment track of *Radil*, which was displayed using Integrative Genomics Viewer (Robinson et al., 2011). (E) Two genes, *Radil* and *Trps1*, from the self-renewal screening candidate gene list, were chosen for the investigation of their function in the maintenance of pluripotency-related markers. Cell lines lacking one of the selected genes were differentiated in N2B27 for 72 h, and the relative expression levels of pluripotent markers *Oct4*, *Sox2*, *Nanog*, and *Rex1* were assessed using qRT-PCR. Unpaired two-tailed student’s *t*-test was used for statistical analysis. Error bars represent mean ± SD. *P < 0.05; **P < 0.01; ***P < 0.001. (F) Inserted genes from puromycin screening were isolated and displayed via scatter plots. Red plots are the genes which related to transportation across the cell membrane. The concentration of puromycin was 0.5 μg/mL, and screening lasted for 7 days. (G) GO analysis of trapped genes from puromycin screening

Through overlapping of trapped genes from three independent libraries, we obtained a gene list that delay the downregulation of pluripotent markers of ESCs (Fig. S2B and Table S1). Furthermore, gene ontology (GO) analysis of trapped genes appeared in 3 libraries revealed that several genes were related to development and cell differentiation (Fig. 2B).

Considering the gene lists from 3 libraries, we identified many genes that participated in the cell fate commitment (Fig. 2B). Although a few genes appeared in each library individually, many genes are shared more than two libraries (Figs. 2C and S2B). For the genes shared in three libraries, we identified *Hira* and *Jarid2*, which was previously implicated in process of ESC differentiation (Fig. 2C) (Leeb et al., 2014). In addition, sixteen of the candidate genes found in our screening have also been identified in a similar study (Leeb et al., 2014) (Table S1). Notably, mutation of *Radil* was recovered in three libraries and had a number of trapped sites (Fig. 2C and 2D), which implied an obstacle in differentiation of ESCs with deficient *Radil*. Moreover, *Radil* and *Trps1* which came from three libraries were selected to identify their function in regulating pluripotency-related genes (Table S1). We knocked out these genes respectively in mice diploid ESCs using CRISPR-Cas9 (Fig. S2C), and then cultured them in suspension with N2B27 medium. After 72 h, embryoid bodies were harvested and pluripotent genes were quantified by quantitative real-time polymerase chain reaction (qRT-PCR). The results showed that *Radil and Trps1* could prevent downregulation of pluripotent genes while in the differentiation medium (Fig. 2E).

Additionally, we set out genes that are resistant to puromycin by performing trapping screening in “One-Shot” haESCs. Compared to self-renewal screening, we obtained a more refined gene list in puromycin screening, which demonstrated that the regulation of self-renewal is more complicated (Table S2). More intriguingly, we also identified a few genes related to drug transportation in puromycin screening (Fig. 2F) (Liang et al., 2015). Moreover, GO analysis of puromycin resistant genes revealed that there was a significant enrichment in the terms of intracellular signal transduction, endocytosis, and components of the membrane (Figs. 2G and S2D). From this part, *Lrp6* was chosen to evaluate its function in the resistance of puromycin. After the knockout of *Lrp6*, cells were cultured in the presence of puromycin for 7 days, and we found that a few colonies survived (Fig. S2E).

In conclusion, we have generated a “One-Shot” screening technique based on PB transposon system in both haESCs and haNSCLCs, and we have established that regardless of the presence or absence of transposition, only one footprint of trapping element was present in a cell. Unexpectedly, we found it was very difficult to completely self-inactivate PBase with 4-OHT treatment. This may be due to the incomplete cutting of “CAGGS” from the Slingshot. Therefore, 4-OHT induced self-inactivation should be optimized in the future. Alternatively, 4-OHT should be supplemented in the medium during the duration of the screening to improve the efficiency of the self-inactivation of PBase. With the help of this screening system, we have isolated functional genes involved in the maintenance of pluripotency-related genes and puromycin resistance in haESCs. Comparing to exist screening system, “One-Shot” haESCs (or haNSCLCs) can be expanded in any capacity homogeneously before induction, and differentiated into different types of haploid somatic cell, which tremendously simplifies the preparation of screening library. Characterized by one mutation per cell, simple library preparation, sequence independence, and lower false positive rate, our “One-Shot” screening system can be employed in numerous processes such as screening of essential genes, targets identification of related drugs, and further screenings in haploid somatic cells. Specially, trapping element in “One-Shot” system can be replaced by other cis-acting elements such as CTCF binding site, and then used for screening which cannot be done in loss of function screening methods. Additionally, although a haploid neural cell library harboring genome-wide mutations has been previously used for the screening of Mn^2+^-mediated toxicity (He et al., 2017), using the “One-Shot” haNSCLCs will simplify the high throughput screening of progenitor or somatic cells for the identification of drug targets or for toxicity studies in the neural system considerably.

## Electronic supplementary material

Below is the link to the electronic supplementary material.

Supplementary material 1 (PDF 499 kb)

Supplementary material 2 (XLSX 93 kb)

Supplementary material 3 (XLSX 18 kb)

Supplementary material 4 (XLSX 11 kb)
