# Supplementary material for: Rapid construction of a whole-genome mutant library by combining haploid stem cells and inducible self-inactivating PiggyBac transposon
Source: Protein Cell. 2020 Mar 19;11(6):452–7. doi: 10.1007/s13238-020-00702-0 (PMC7251005; doi:10.1007/s13238-020-00702-0)
Supplement: Supplementary file 1 — Supplementary material 1 (PDF 499 kb) [file 13238_2020_702_MOESM1_ESM.pdf]

Supplementary material

Materials and Methods

Plasmid construction

Plasmid Slingshot was obtained from DAVID (Kong et al., 2010), and splice acceptor-IRES, promoterless β-geo in Slingshot was replaced by splice acceptor-IRES-Neo. Additionally, homologous arms of mRosa26 were cloned into upstream of 5' TR and downstream of selection biomarker BSD. To prevent the effect of *mRosa26* locus, poly A element was inserted between homologous arm and 5' TR. Another element, PGK-TK-PA was placed outside the 3' homologous arm to prevent random insertion, and final plasmid OS-TK was sequenced in full.

haESCs culture and "One-Shot" screening system generation

haESCs derived from mice androgenetic haploid blastocysts (Li et al., 2012) were cultured in N2B27 medium supplemented with 1 mM PD0325901 (MEK inhibitor; Stemgent, 04-0006), 3 mM CHIR99021 (GSK3b inhibitor; Stemgent, 04-0004), and 20 ng/mL LIF (Millipore, ESG1107) (Ying et al., 2008), and haploid cells were purified using fluorescence-activated cell sorting (FACS) for every 10 passages. To generate "One-Shot" carrying haESCs, plasmids of 10 μg OS-TK, 5 μg Rosa26 specified sgRNA, and 5 μg Cas9 were co-electroporated into 4×10^6 haESCs, and a few clones appeared after 7 days selection with 250 ng/mL geneticin (Gibco, 11811-031) and 8 μM ganciclovir (Invivogen, sud-gcv). Then, these clones were picked up and analyzed using PCR.

Generation of haNSCLCs

"One-Shot" haNSCLCs were differentiated from "One-Shot" haESCs (He et al., 2017). Briefly, "One-Shot" haESCs were cultured in suspension for embryoid Body (EB) formation for 3 days and were plated onto dishes coated with fibronectin (Gibco, G1890). The medium in this step was haNSCLCs optimized medium, which consists of N2B27 medium supplemented with 20 ng/mL FGF2 (R&D Systems, 233-FB-001MG/CF), 20 ng/mL EGF (R&D, 2028-EG-200), 300 nM LDN193189 (MedChemExpress [MCE], HY-12071A), 10 μM SB431542 (Stemgent, 04-0010-10), and 20 μM Y-27632 (MedChemExpress [MCE], Y-27632). About 5 days later, most

of cells expanded from EBs and proliferated with monolayer. Cells from this stage were dissociated into single cell and plated onto poly-D-lysine (10 μg/mL; Sigma, P6407) and laminin (10 μg/mL; Invitrogen, 23017-015) coated dish, and then cultured in haNSCLCs optimized medium for another 4 days. At day 10 after initial differentiation, the differentiated cells were dissociated for DNA content analysis using Hoechst 33342 (2.5 μg/mL; ThermoFisher, H3570).

Immunofluorescence staining

Cells were fixed with 4% paraformaldehyde (Sigma, P6148-1KG) at room temperature for 30 min, and then blocked in 2% BSA (Sigma, A3311-100g) for 1 h. Primary antibodies anti-Erα (used to locate PBase; SANTA CRUZ, sc-7207), anti-SOX2 (Millipore, AB5603) and anti-NESTIN (Millipore, MAB353) were diluted in 2% BSA and incubated at 4°C overnight.

Southern blotting

After PCR analysis, 3 clones were selected for Southern blotting. Genomes of the clones were extracted using kit (MicroElute Genomic DNA Kit, Omega, D3096-01), and then 30 μg genomes of each clone were digested with KpnI (New England Biolabs, R3142L) and AgeI (New England Biolabs, R3552L) for 16 h. The digested genomes were fractionated in 0.8% agarose gels and were analyzed using standard Southern blotting procedure. The probe in this part was a 399 bp fragment amplified from OS-TK plasmid. Primers of probe are listed in Table S3.

Splinkerette PCR

Splinkerette PCR contained three steps, genome digestion, adaptor junction, and nested PCR amplification (Horn et al., 2007). For the first step, 0.5 μg of genomes were digested at 37°C with BstYI (New England Biolabs, R0523L) for 16 h, and then the endonuclease was inactivated at 80°C for 30 min. The inactivated digested genomes were then ligated with annealed splinkerette adapter at 4°C for 16 h. The ligation mixture was used as template for nested PCR, with primers designed on both PB terminal repeats and splinkerette adapter. Sequences of adapter and primers are listed in Table S3.

Genome coverage evaluation

For genome coverage evaluation, 4×10^7 cells (haESCs and haNSCLCs respectively) were

induced to trap the genome, and the inserted sites were isolated using splinkerette PCR and high throughput sequencing. The second round of the nested PCR primers was used as barcodes to filter reads. We then extracted the sequences contained 'TTAA' using cutadapt (Martin, 2011), and then aligned them to mouse reference genome from ensembl release 97 using bwa aligner (Li and Durbin, 2010). PCR duplications in alignments were removed using gatk (McKenna et al., 2010), and cleaned alignments were used to count reads with the window of 100,000 bp using deeptools2 (Ramirez et al., 2016) and diagrammed via circos (Krzywinski et al., 2009), genome features enrichment of reads were analyzed using ALFA (Bahin et al., 2019).

Insert sites annotation

Firstly, reads were filtered or demultiplexed using cutadapt, and then aligned to mouse reference genome via bwa described above. Strand orientation of alignments and trapping events was analyzed by bedtools (Quinlan and Hall, 2010) and Linux scripts. "Sense Reads" represented reads that have the same orientation as the trapping element of the "One-Shot" system and genes. "Sense rate" and "dispersion" were calculated using the following formulas:

$$sense\ rate = \frac{sense\ reads}{total\ reads}$$

$$dispersion = sense\ rate - 0.5$$

Trapped genes (with total reads more than 50) were featured using DAVID bioinformatics resources (Huang da et al., 2009). All duplications in this analysis were removed using gatk toolkits.

Barcodes encoded splinkerette PCR

Fifteen barcodes were designed, and barcodes sequences are listed in Table S3. For single clone inserted sites isolation, inserted sites of each clone were isolated using splinkerette PCR and then mixed for sequencing via high throughput sequencing. Cleaned Raw data was demultiplexed and mapped to the mouse reference genome, and alignments without PCR duplications were counted with the window of 100,000 bp. Windows were then sorted by the number of alignments, and top 5 windows for each clone were shown using scatter plots.

Differentiation screening

"One-Shot" Rex1-GFP haESCs (OsRG-haESCs) were purified by FACS and expanded for the population of 4.5×10^7. These OsRG-haESCs were divided into 3 libraries and cultured in

suspension with 4-Hydroxytamoxifen for 3 days to form mutated EBs in the differentiation medium (N2B27 medium without 2i and LIF), and then were plated onto fibronectin (Gibco, G1890) coated dishes. After 32 days of differentiation, GFP positive cells in each library were obtained using flow cytometry respectively. Two libraries were sequenced and analyzed directly, and GFP positive cells from the third library were plated onto feeder coated dish in N2B27 medium supplemented with 2i and LIF for expansion. And then, inserted sites of GFP positive colonies from the third library were isolated using splinkerette PCR and high throughput sequencing. Genes with more than 50 reads were considered as candidates for maintenance of self-renewal.

Puromycin screening

"One-Shot" haESCs were purified for haploid before puromycin screening, and 4.5×10^7 "One-Shot" haESCs were treated with 4-OHT for 3 days to form mutated library. Mutated cells were cultured in ESC medium with puromycin (0.5 μg/mL; Gibco, A1113803) for 7 days, and then survived cells were dissociated to identified inserted sites using splinkerette PCR and high throughput sequencing. For puromycin screening, genes with at least 20 reads were treated as candidates.

Quantitative real-time polymerase chain reaction (qRT-PCR)

Total RNA from cells was extracted using PureLink™ RNA Mini Kit (Invitrogen, 12183018A), and 2 μg of total RNA was used to synthesize complementary DNA via M-MLV Reverse Transcriptase (Promega, M1705) according to instruction. Then proper cDNA was added to SYBR Green Realtime PCR Master Mix (TOYOBO, QPS-201) based qRT-PCR volume to evaluate the abundance. All samples were utilized in triplicates, and data were normalized to housekeeping gene *Gapdh* and analyzed with delta-delta Ct analysis. Primers used for qRT-PCR were listed in Table S3.

AP staining

AP staining was in accordance with the steps provided in alkaline phosphatase kit (Beyotime, C3206).

References

Bahin, M., Noel, B.F., Murigneux, V., Bernard, C., Bastianelli, L., Le Hir, H., Lebreton, A., and Genovesio, A. (2019). ALFA: annotation landscape for aligned reads. BMC Genomics *20*, 250.

He, Z.Q., Xia, B.L., Wang, Y.K., Li, J., Feng, G.H., Zhang, L.L., Li, Y.H., Wan, H.F., Li, T.D., Xu, K.*, et al.* (2017). Generation of Mouse Haploid Somatic Cells by Small Molecules for Genome-wide Genetic Screening. Cell reports *20*, 2227-2237.
Horn, C., Hansen, J., Schnutgen, F., Seisenberger, C., Floss, T., Irgang, M., De-Zolt, S., Wurst, W., von Melchner, H., and Noppinger, P.R. (2007). Splinkerette PCR for more efficient characterization of gene trap events. Nature genetics *39*, 933-934.
Huang da, W., Sherman, B.T., and Lempicki, R.A. (2009). Systematic and integrative analysis of large gene lists using DAVID bioinformatics resources. Nat Protoc *4*, 44-57.
Kong, J., Wang, F., Brenton, J.D., and Adams, D.J. (2010). Slingshot: a PiggyBac based transposon system for tamoxifen-inducible 'self-inactivating' insertional mutagenesis. Nucleic acids research *38*, e173.
Krzywinski, M.I., Schein, J.E., Birol, I., Connors, J., Gascoyne, R., Horsman, D., Jones, S.J., and Marra, M.A. (2009). Circos: An information aesthetic for comparative genomics. Genome research.
Li, H., and Durbin, R. (2010). Fast and accurate long-read alignment with Burrows–Wheeler transform. Bioinformatics *26*, 589-595.
Li, W., Shuai, L., Wan, H., Dong, M., Wang, M., Sang, L., Feng, C., Luo, G.Z., Li, T., Li, X.*, et al.* (2012). Androgenetic haploid embryonic stem cells produce live transgenic mice. Nature *490*, 407-411.
Martin, M. (2011). CUTADAPT removes adapter sequences from high-throughput sequencing reads, Vol 17.
McKenna, A., Hanna, M., Banks, E., Sivachenko, A., Cibulskis, K., Kernytsky, A., Garimella, K., Altshuler, D., Gabriel, S., Daly, M.*, et al.* (2010). The Genome Analysis Toolkit: a MapReduce framework for analyzing next-generation DNA sequencing data. Genome research *20*, 1297-1303.
Quinlan, A.R., and Hall, I.M. (2010). BEDTools: a flexible suite of utilities for comparing genomic features. Bioinformatics *26*, 841-842.
Ramirez, F., Ryan, D.P., Gruning, B., Bhardwaj, V., Kilpert, F., Richter, A.S., Heyne, S., Dundar, F., and Manke, T. (2016). deepTools2: a next generation web server for deep-sequencing data analysis. Nucleic acids research *44*, W160-165.
Ying, Q.L., Wray, J., Nichols, J., Batlle-Morera, L., Doble, B., Woodgett, J., Cohen, P., and Smith, A. (2008). The ground state of embryonic stem cell self-renewal. Nature *453*, 519-523.

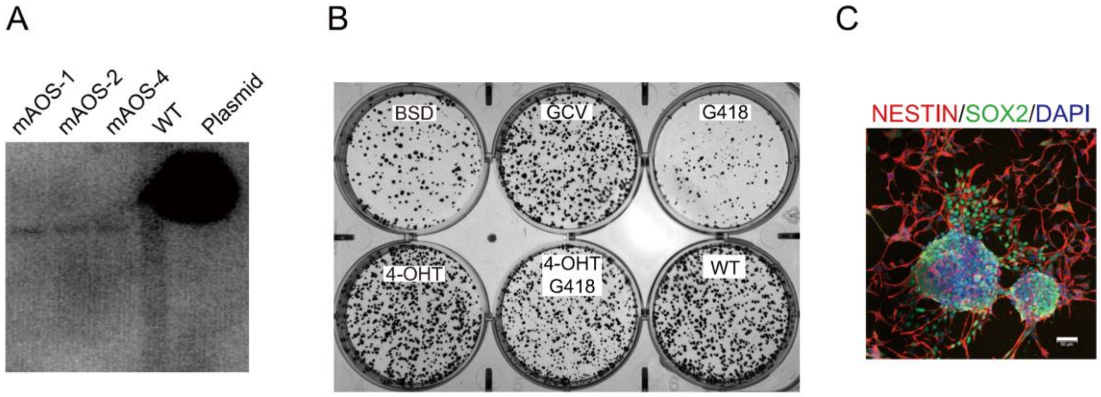

Supplementary Figure S1. Generation of "One-Shot" system in haESCs and haNSCLCs

(A) Southern analysis shows copy number of trapping cassette in "One-Shot" screening system. Genomes were digested with KpnI (New England Biolabs, R3142L) and AgeI (New England Biolabs, R3552L). (B) Drugs resistance analysis of "One-Shot" haESCs, and the recipe included blasticidin (15 μg/mL), GCV (8 μM), G418 (250 μg/mL), and 4-OHT (1 μM). (C) Immunostaining of neural stem cell specific marker genes *Sox2* and *Nestin* in "One-Shot" haploid neural stem cell-like cells (haNSCLCs). Nuclei were stained with DAPI. "One-Shot" haNSCLCs were differentiated from "One-Shot" haESCs with the protocol we used previously (He et al., 2017), scale bar, 50 μm.

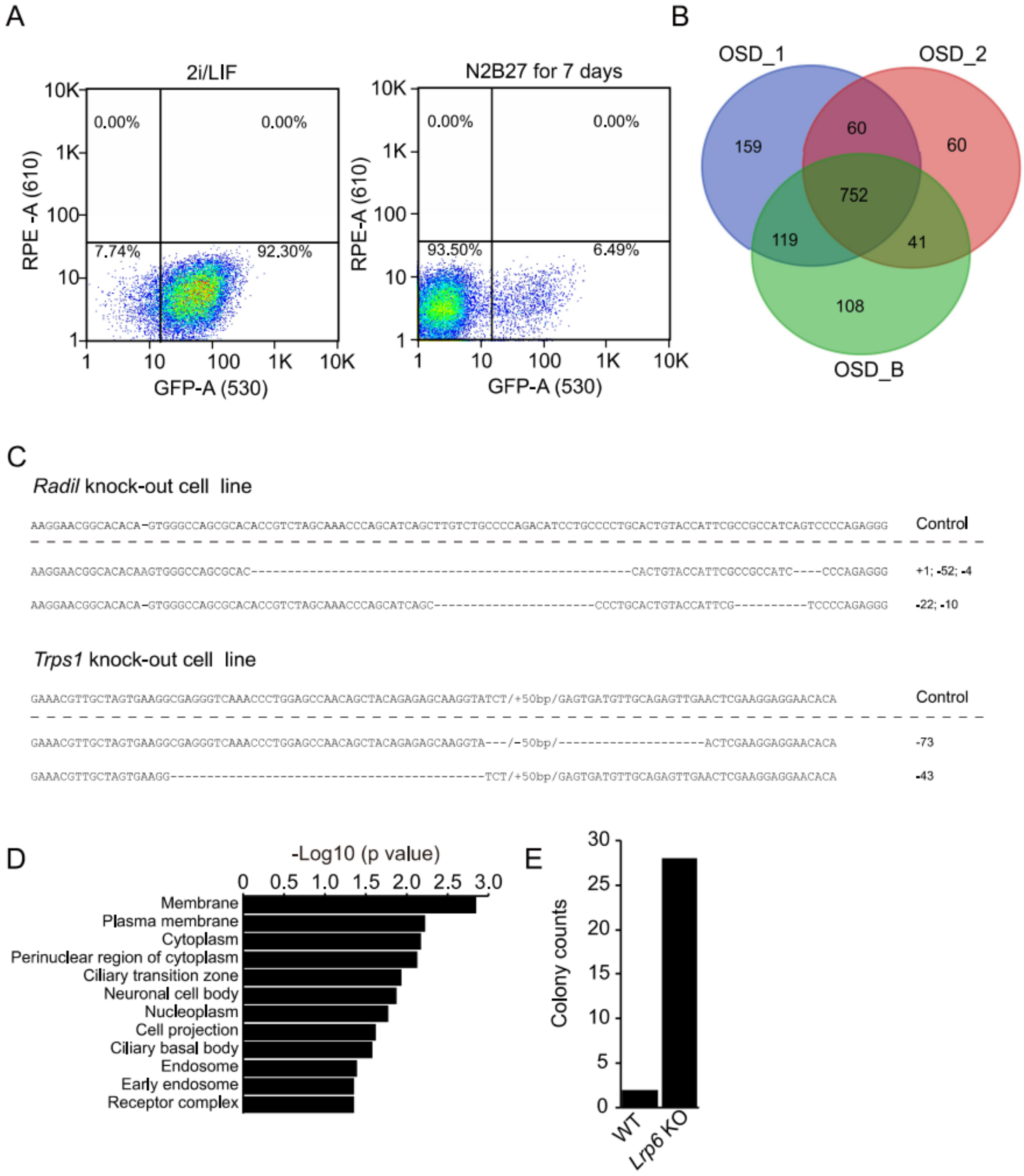

Supplementary Figure S2. Self-renewal and puromycin screening using "One-Shot" system

(A) Rex1-GFP maintenance during differentiation. Flow cytometry demonstrated the expression of Rex1-GFP before differentiation in 2i/LIF and differentiation for 7 days in N2B27. (B) Trapped genes in three independent libraries for self-renewal screening. Venn diagram showed genes sharing for 3 libraries or specific for each library. (C) The genotype of the *Radil* and *Trps1* knock-out cell lines. For Trps1, +50 bp means that there are 50 base pairs in the wildtype cell line at that position, and -50 bp means that 50 base pairs are missing in the knock-out cell line. (D) Gene ontology (GO) analysis for cell components of trapped genes from puromycin screening. (E) Statistic of colonies of WT cells and *Lrp6* deficient cells, *Lrp6* knockout resulted in puromycin resistance.
